# Synergistic drug interactions of the histone deacetylase inhibitor givinostat (ITF2357) in CRLF2-rearranged pediatric B-cell precursor acute lymphoblastic leukemia identified by high-throughput drug screening

**DOI:** 10.1016/j.heliyon.2024.e34033

**Published:** 2024-07-03

**Authors:** Athanasios Oikonomou, Titus Watrin, Luigia Valsecchi, Katerina Scharov, Angela Maria Savino, Julian Schliehe-Diecks, Michela Bardini, Grazia Fazio, Silvia Bresolin, Andrea Biondi, Arndt Borkhardt, Sanil Bhatia, Giovanni Cazzaniga, Chiara Palmi

**Affiliations:** a Tettamanti Center, Fondazione IRCCS San Gerardo dei Tintori, Monza, Italy; b Department of Paediatric Oncology, Haematology and Clinical Immunology, Heinrich-Heine University Dusseldorf, Medical Faculty, Düsseldorf, Germany; c School of Medicine and Surgery, University of Milano-Bicocca, Italy; d Pediatric Hematology, Oncology and Stem Cell Transplant Division, Women and Child Health Department, Padua University and Hospital, Padua, Italy; e Onco-Hematology, Stem Cell Transplant and Gene Therapy, Istituto di Ricerca Pediatrica Foundation - Città della Speranza, Padua, Italy; f Pediatrics, Fondazione IRCCS San Gerardo dei Tintori, Monza, Italy

**Keywords:** High-throughput drug screening, CRLF2 rearranged acute lymphoblastic leukemia, Combination treatment, Givinostat, Trametinib, Venetoclax

## Abstract

Combining multiple drugs broadens the window of therapeutic opportunities and is crucial for diseases that are currently lacking fully curative treatments. A powerful emerging tool for selecting effective drugs and combinations is the high-throughput drug screening (HTP). The histone deacetylase inhibitor (HDACi) givinostat (ITF2357) has been shown to act effectively against CRLF2-rearranged pediatric B-cell precursor acute lymphoblastic leukemia (BCP-ALL), a subtype characterized by poor outcome and enriched in children with Down Syndrome, very fragile patients with a high susceptibility to treatment-related toxicity. The aim of this study is to investigate possible synergies with givinostat for these difficult-to-treat patients by performing HTP screening with a library of 174 drugs, either approved or in preclinical studies. By applying this approach to the CRLF2-r MHH-CALL-4 cell line, we identified 19 compounds with higher sensitivity in combination with givinostat compared to the single treatments. Next, the synergy between givinostat and the promising candidates was further validated in CRLF2r cell lines with a broad matrix of concentrations. The combinations with trametinib (MEKi) or venetoclax (BCL2i) were found to be the most effective and with the greatest synergy across three metrics (ZIP, HAS, Bliss). Their efficacy was confirmed in primary blasts treated *ex vivo* at concentration ranges with a safe profile on healthy cells. Finally, we described givinostat-induced modifications in gene expression of MAPK and BCL-2 family members, supporting the observed synergistic interactions. Overall, our study represents a model of drug repurposing strategy using HTP screening for identifying synergistic, efficient, and safe drug combinations.

## 1 Introduction

Due to the complex interplay between genetics, molecular processes, and environmental factors in cancer cells, the one-target one-drug strategy frequently falls short of achieving complete pharmacological inhibition [1]. Cancer cells are frequently able to escape the pressure of a single drug and treatment-resistant subpopulations often emerge [1,2]. Moreover, the multi-targeting approach allows the establishment of an optimal balance between efficacy and toxicity. Indeed, thanks to the synergistic or additive pharmacodynamic drug-drug interaction, the doses of one or more drugs can be reduced, leading to fewer side effects. On the other hand, it offers the opportunity of targeting diverse or inter-connected pathological pathways at the same time, avoiding the generation of resistant clones [3]. Therefore, the application of multi-agent regimens is a promising anti-cancer therapeutic strategy and the identification of synergistic drug interactions is necessary to achieve this [4].

Pediatric acute lymphoblastic leukemia (ALL) was the first disease where a combination treatment was successfully implemented, with initial drug combinations developed in the 1960s forming the basis of combination therapy which has continued to be iteratively improved since that time [4]. This approach paved the way for further research and clinical application of combinations of chemotherapeutics also in other diseases [5]. Identifying effective drug combinations from a vast pool of compounds requires a rational approach that integrates multidisciplinary knowledge to prioritize interactions with the highest efficacy, considering disease pathogenesis and drug mechanisms [6,7].

For this purpose, high-throughput (HTP) preclinical drug testing on cancer cell lines and patient samples has recently started to be used as a rational large-scale assay for the discovery of successful drug combinations [[8], [9], [10]], bridging functional and computational approaches [11,12]. Interestingly, by selecting drugs that are already FDA/EMA-approved for the HTP screening, the window of therapeutic candidates can be further narrowed. Several pieces of evidence indicate that drugs already proven to be effective in a certain context can be reused in a new setting where the clinical needs are unmet [13].

B-cell precursor ALL (BCP-ALL) is the most frequent malignancy in children and despite at present the cure rate approaching 90 %, the probability of survival ranges between 30 and 50 % for patients with relapse [14]. Therefore, novel therapeutic strategies, especially for poor prognosis patient subgroups, are needed. Rearrangements involving *cytokine receptor-like factor 2* (*CRLF2)* gene (CRLF2r) have been identified in approximately 10 % of pediatric BCP-ALL [15], but their incidence increases to approximately 50 % in children with Philadelphia-like (Ph-like) BCP-ALL [16], and in Down syndrome (DS)-associated BCP-ALL [17,18]. Ph-like BCP-ALL is a subgroup that shares a similar gene expression profile (GEP) with patients positive for *BCR::ABL* fusion [19]. These two specific subgroups of patients are characterized by a high risk of relapse [20], and particularly for DS patients by an increased susceptibility to treatment-related toxicity [21].

*CRLF2* gene encodes for a component of the heterodimeric thymic stromal lymphopoietin (TSLP) receptor. The chromosomal abnormalities involving *CRLF2* gene (*P2RY8::CRLF2* and *IGH@::CRLF2*) cause the overexpression of this receptor, hyperactivation of JAK/STAT and PI3K/mTOR pathways and are often associated with mutations in *JAK2* gene and with the deletion of *IKZF1* gene [[22], [23], [24]]. Moreover, the co-occurrence of *P2RY8::CRLF2* gene fusion and *IKZF1* deletion determines a condition called “IKZF1plus" which characterizes DS and non-DS patients with a dismal prognosis [25,26]. Therefore, new, more effective and less toxic therapeutic options are a formidable challenge for the CRLF2r BCP-ALL subgroup.

In our previous work, we identified a pan-histone deacetylase (HDAC) inhibitor, givinostat (ITF2357), which effectively downregulates CRLF2r BCP-ALL [27]. This drug causes inactivation of JAK/STAT signaling network and induces *in vitro*, *ex vivo*, and *in vivo* leukemic cell death, sparing the normal hematopoietic counterpart [27]. Moreover, we proved that givinostat was able to kill tumor cells resistant to the JAK1/2 kinase inhibitor ruxolitinib which is currently being investigated in children with JAK/STAT5-activated leukemias [28,29].

The pharmacological potential of givinostat was initially focused on its anti-inflammatory capacity [30], with its first anti-tumor evidence reported in multiple myeloma and acute myeloid leukemia (AML) [31]. Afterwards, this compound has been shown as highly effective against malignancies carrying mutations of JAK2, which leads to a hyperactivation of JAK/STAT pathway [32], and it is currently undergoing clinical trials in polycythemia vera [33] and other JAK2V617F positive myeloproliferative neoplasms, such as essential thrombocythemia and primary myelofibrosis [34]. Moreover, givinostat has been proven to be potent in other hematological malignancies beyond myeloproliferative neoplasms [35], further extending its indication to juvenile idiopathic arthritis [36] and Duchenne muscular dystrophy [37], where it is currently approved by FDA and has started the regulatory review process of EMA.

Overall, the pharmacological profile of givinostat makes it an efficient, safe, and well-tolerated compound, as demonstrated by both pre-clinical and clinical observations, representing an ideal drug for the therapy of pediatric CRLF2r BCP-ALL patients [27]. Therefore, in this study we investigated the potential synergistic interactions of givinostat with other drugs, in order to find the most appropriate therapeutic strategy for these difficult-to-treat patients, utilizing a HTP drug screening repurposing approach to dissect promising drug candidates as combination partners.

## 2 Materials and methods

### 2.1 High-throughput drug screening and analysis

**Fig. 1:**
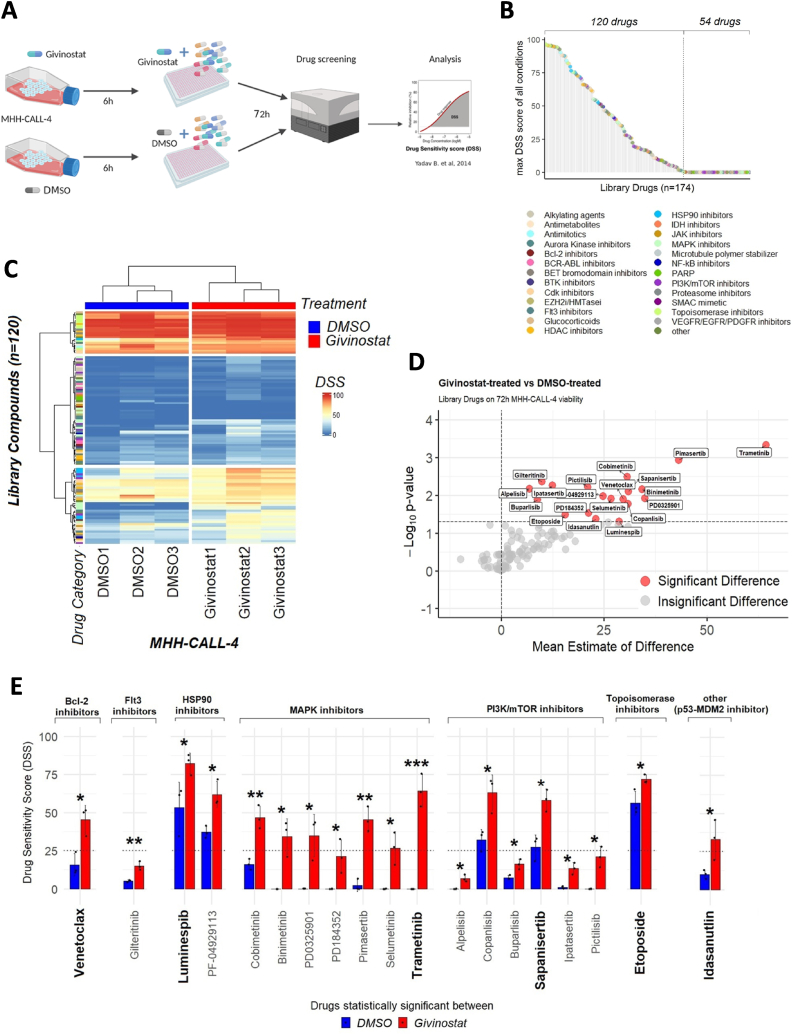
Givinostat combination high-throughput drug screening on MHH-CALL-4. A. Schematic illustration summarizing the experimental workflow performed. B. Plot showing the maximum DSS for the 174 library compounds across all conditions evaluated (replicates of givinostat- and DMSO-treated MHH-CALL-4) sorted from the highest to the lowest and color-coded according to the drug categories. C. Hierarchical clustering heatmap analysis of the 120 library drugs with a non-zero maximum DSS, across the tested conditions on MHH-CALL-4. Color intensity reflects the DSS. For the drug category colors see the legend of Fig. 1B. D. Volcano plot summarizing the statistical significance between the DMSO versus givinostat treatment on MHH-CALL-4 evaluated by the two-sample *t*-test with equal variance of compounds' sensitivity (DSS). Compounds highlighted in red (n = 19, annotated) possess significantly higher sensitivity in the givinostat-treated condition (p-value<0.05). E. Bar plot illustrating the DSS score of DMSO- (blue) and givinostat-treated (red) MHH-CALL-4 for the 19 compounds with statistically higher sensitivity upon givinostat. Compounds are gathered based on their drug category. The threshold of 25 DSS is indicated. The 6 drugs selected for following analyses are highlighted in bold. Data are presented as Mean with Standard Deviation. Two-sample *t*-test with equal variance was applied (*p < 0.05, **p < 0.01, ***p < 0.001; n = 3 replicates per condition). (For interpretation of the references to color in this figure legend, the reader is referred to the Web version of this article.)

High-throughput Drug Screening was performed as previously described [26,38]. Briefly, 1536-well microplates were pre-coated in a randomized way by Tecan d300 Dispenser (Tecan, Männedorf, Switzerland) with a custom library comprising 174 compounds FDA/EMA-approved or in preclinical studies (purchased from Selleckchem, TX, USA or MedChem Express, NJ, USA). Six different concentrations for each drug were included, ranging from 8 nM to 25 μM. The DMSO vehicle and the cell death inducer staurosporin were used as negative and positive controls, respectively. The CRLF2r BCP-ALL cell line MHH-CALL-4, pre-treated for 6 h with DMSO or givinostat at 0.05 μM, was seeded in three independent compound-pre-coated microplates (biological triplicates) by Thermo Multidrop reagent Dispenser (Thermo Fisher Scientific) at the concentration of 0.5 × 10^6^ cells/mL. Givinostat at 0.05 μM or DMSO was added again in the medium of the cells during the exposure to the library drugs. After 3 days, the cell viability was evaluated using the Cell Titer Glo (CTG) assay (Promega, Madison, USA) on SPARK 10 M microplate reader (Tecan), according to the manufacturer's instructions, by measuring the ATP production as an estimate of the metabolic activity of cells. For each drug, a quantitative drug sensitivity score (DSS) was computed as previously described [39] (Fig. 1A). Unsupervised hierarchical heatmap analysis was performed in R Studio (package “pheatmap”) using the DSS values of each sample for compounds with non-zero activity across the whole dataset. Two-sample *t*-test with equal variance was used to evaluate the difference in sensitivity of compounds upon treatment with givinostat versus DMSO. A compound was considered effective for givinostat condition when the mean DSS was higher than 25, excluding in this way the compounds with activity at the lower quartile of the DSS scale (0,100). Statistics and data visualization were performed in R Studio using the packages “matrixTests”, “Enhanced Volcano” and “ggplot2” respectively.

### 2.2 Cell lines and patient samples

The CRLF2r BCP-ALL cell lines used consisted of MHH-CALL-4 and MUTZ-5, both overexpressing *CRLF2* via *IGH@::CRLF2* translocation and harboring JAK2 mutations (JAK2I682F and R683G, respectively) [22] and *IKZF1* deletions. The cell lines were purchased from DSMZ (Braunschweig, Germany. MHH-CALL4: established from the peripheral blood of a 10-year-old boy with acute lymphoblastic leukemia (pre B-ALL) at diagnosis in 1993, DSMZ catalogue number ACC337, RRID:CVCL_1410; MUTZ5: established in 1998 from the peripheral blood of a 26-year-old man with B cell precursor acute lymphoblastic leukemia (BCP-ALL) at relapse, DSMZ catalogue number ACC490, RRID:CVCL_1873) and were maintained at 37 °C in a humidified atmosphere at 5 % CO_2_ in Advanced RPMI-1640 medium (Thermo Fisher, Waltham, Massachusetts) with the addition of 20 % fetal bovine serum (FBS, Biosera, Cholet, France), 1 % penicillin/streptomycin and 1 % l-glutamine (both from Euroclone, Pero, Italy). The cell line identity was confirmed by short tandem repeat (STR) profiling performed at the Technion Genomics Center, Technion Israel Institute of Technology.

Five pediatric BCP-ALL patients enrolled in the Italian Centers and treated according to the AIEOP-BFM protocols (NCT00613457 and NCT01117441) were selected according to their positivity for *CRLF2* alterations and availability of biological material. Four out of the 5 patients had Down Syndrome (Table 1).

**Table 1:** Clinical and molecular characteristics of the patients.

*Sample no*.	*Protocol*	*Age at diagnosis (years)*	*Sex*	*WBC x* 103/μl	*Down syndrome*	*Immuno phenotype*	*Final risk*	*CRLF2 rearrangement*	*JAK2*	*IKZF1*
Pt #S1 DS	ALL2009	15	M	1870	Yes	B-II	High risk	P2RY8:CRLF2	L681-I682 insGL	del (ex 2–7)
Pt #S5 DS	ALL2000/06	2	M	12900	Yes	B-II	Standard risk	P2RY8:CRLF2	R683G	wt
Pt #S8 DS	ALL2009	2	M	23 940	Yes	B-II	Standard risk	P2RY8:CRLF2	wt	wt
Pt #S9 DS	ALL2000/06	8	M	66960	Yes	B-II	Medium risk	P2RY8:CRLF2	R683G	del (ex 4–7)
Pt #S19	ALL2000/06	5	F	16 600	No	B-II	Standard risk	P2RY8:CRLF2	wt	wt

Abbreviations: Pt, patient; DS, Down Syndrome; F, female; M, male.; wt, wild-type; ins, insertion; del, deletion; ex, exon.

### 2.3 *In vivo* murine xenotransplantation of BCP-ALL primary samples

The diagnostic material, bone marrow (BM) or peripheral blood (PB) cells, from 5 pediatric patients with BCP-ALL was transplanted into the tail vein of immunodeficient NOD.Cg-PrkdcSCIDIl2rgtm1Wjl/SzJ mice (NSG, Charles River, Calco Italy) to generate Patient-derived xenograft (PDX) models and expand the bulk leukemia. When signs of overt leukemia appeared, mice were sacrificed and BM and spleen cells were collected. The leukemia engraftment was assessed by staining with anti-hCD45 (Cat: 555485, BD Biosciences, Franklin Lakes, NJ, USA), anti-hCD19 (Cat: 555414, BD Biosciences or Cat.11-0199, eBiosciences, Thermo Fisher), anti-hCD10 (Cat: 17-0106-42, eBiosciences) and anti-mCD45 antibody (cat. 12–0451, eBioscience), followed by FACS analysis using LSRFortessa X-20 (BD Biosciences). All the BM or spleen samples used for *ex vivo* assays displayed a >90 % leukemic engraftment evaluated by FACS analysis.

### 2.4 Validation of HTP drug combinations

Validation of promising drug combinations resulting from HTP drug screening was primarily carried out on MHH-CALL-4 and MUTZ-5 cell lines and on 3 B-cell lymphoblastoid cell lines (one derived from a pediatric healthy donor [40], two derived from non-leukemic children with Down Syndrome [41]). The compounds for validation were purchased from Selleckchem and MedChemExpress. Treatment of the cell lines was performed on 96-well plates in RPMI Advanced (Gibco, Thermo Fisher Scientific) with 20 % FBS, 1 % Penicillin/Streptomycin (Cambrex, BioScience, Milan, Italy), and 1 % l-Glutamine upon a matrix of increasing concentrations of givinostat and of the second drug. The decrease in cell viability was evaluated after 72h by Cell Titer Glo luminescence assay on a SPARK 10 M microplate reader following the manufacturer's instructions.

The matrix of vehicle-normalized inhibition values was analyzed using “SynergyFinder” package in R [42] to compute metrics such as Zero-Potency Interaction (ZIP), Highest Single Agent (HSA), and Bliss synergy scores, as well as Combination Sensitivity Score (CSS), a parameter reflecting the efficacy achieved under a given synergy for each drug combination [43]. These three synergy metrics were individually plotted against CSS and only the drug combinations consistently observed in the upper right quadrant of the graph (i.e., highly synergistic and effective), across all interaction metrics utilized and in both tested CRLF2r cell lines, were selected for further analysis.

Subsequently, we have generated heatmaps of synergy and dose-response inhibition by “SynergyFinder” and selected the most synergistic concentration ranges according to the ZIP model. This provided a reference for guiding drug combination treatments in PDX samples, where the low availability of cells restricts the possibility of multiple experimental conditions. Given this limitation, we assessed the degree of interaction using a fixed dose of givinostat, trametinib, and venetoclax in PDX samples using the Bliss model.

Five BCP-ALL PDX samples (Table 1) were used to validate the efficacy of the combination of givinostat + venetoclax and givinostat + trametinib treatments *ex vivo* in independent experiments. The BM cells or splenocytes collected from mice with >90 % leukemic engraftment were seeded at the concentration of 2.4 × 10^6^ cells/ml in serum-free StemSpan (Stemcell Technologies, Vancouver, Canada) supplemented with 1 % GlutaMAX (Gibco, Thermo Fisher Scientific), 1 % Penicillin/Streptomycin, 10 ng/mL human recombinant SCF and 10 ng/mL human recombinant G-CSF (Peprotech, London, UK) in absence of drugs (DMSO) or in presence of the above-mentioned drugs as single or combination treatment. The cell viability was evaluated following 72h of treatment by FACS analysis of apoptotic cells after AnnexinV/7-AAD staining (Enzo Life Science, Lausen, Switzerland). Drug additivity or synergy was determined, as mentioned above, using the Bliss score of drugs independence [44], calculated with the formula: E_ab_ = E_a_ + E_b_-(E_a_*E_b_), where Eab is the additive effect of drugs “a” and “b” as predicted by their observed individual effects (E_a_ and E_b_). Therefore, E_ab_ represents the expected value (EV) in case of additivity of the compounds, while the observed value (OV) indicates the actual combination effect. Based on this formula, drugs are additive when OV = EV, synergistic when OV > EV, and antagonistic when OV < EV. The statistical significance of the difference between EV and OV was evaluated by Welch's unpaired *t*-test. Statistics and data visualization were performed in GraphPad Prism6 (GraphPad Software, Inc.).

### 2.5 Microarray gene expression analysis

Microarray raw data (CEL files) and probe set signals of our study GSE77270 [27], available at the National Center for Biotechnology Information Gene Expression Omnibus database (GEO, http://www.ncbi.nlm.nih.gov/geo/), were downloaded and re-analyzed. This series contains gene expression profiles measured using the Affymetrix platform (AffymetrixGeneChip Human Genome U133 Plus 2.0 array and the AffymetrixGeneChip 3’ IVT PLUS reagent kit) of 5 CRLF2r BCP-ALL patient-derived xenograft samples treated with vehicle (DMSO) or givinostat *ex vivo* at 0.2 μM for 6 h. Three out of the five patients of this publicly available dataset (Pt #1, #3 and #4; [27]) are the same patients used for the *ex vivo* validation of the givinostat combinations in this current study (Pt #1 is Pt #S19, Pt #3 is Pt #S8 DS and Pt #4 is Pt #S1 DS). Re-analysis of the data was performed as previously described [27], extracting the affymetrix probes with statistically significant (adjusted p-value as false discovery rate (FDR) < 0.05) differential expression between the two conditions. We then dissected the genes that are known or predicted to interact with trametinib or with venetoclax according to the Drug-Gene Interaction database (DGIdb) [45], comprising both known and unknown interaction types and directionality (inhibitor, antagonist, agonist, others). Multiple affymetrix probes corresponding to the dissected genes were collapsed to single genes using the Gene Set Enrichment Analysis (GSEA) software [46] and its “CollapseDataset” function, setting collapsing mode to max probe. Expression of all DGIdb-annotated trametinib and venetoclax interacting genes was visualized in a heatmap (R Studio package “pheatmap”). Similarly, we also verified the expression of genes belonging to BCL-2 family, considering their affymetrix probe sets as previously summarized [47], in DMSO- versus givinostat-treated samples to identify those members with deregulated profiles upon givinostat. Finally, we performed KEGG enrichment analysis for all the differentially expressed genes of the GSE77270 study using “pathfindR” package in R [48]. Data visualization was carried out using the R package “ggplot2”.

### 2.6 Graphical representation

Biorender software was used for the schematic representation of the experimental workflows performed in this study (BioRender.com).

## 3 Results

### 3.1 Combinatorial HTP drug screening on MHH-CALL-4 identified compounds whose sensitivity was affected by givinostat

As we have previously identified givinostat as a promising anti-leukemic agent for CRLF2r BCP-ALL cases [27], we sought to evaluate its synergistic capability with other compounds FDA/EMA-approved or in preclinical studies included in a HTP drug screening library of 174 compounds [26]. Using the MHH-CALL-4 cell line as a model, we constructed a per-drug DSS score to unravel compound sensitivity in givinostat and DMSO condition (Fig. 1A).

As shown in Figs. 1B, 54 compounds presented no activity in MHH-CALL-4 while the remaining 120 compounds were used for further investigation. Interestingly, the unsupervised hierarchical clustering heatmap analysis, based on these 120 drugs, was able to distinguish givinostat- and DMSO-treated MHH-CALL-4 (Fig. 1C). We observed 3 major clusters of compounds with one (Fig. 1C at the bottom) comprising different drugs belonging to various categories, whose sensitivity was more modulated by givinostat. To evaluate the statistical significance of the difference in compound sensitivity between the two treatments, we applied the equal variance *t*-test and identified 19 compounds with a statistically significant higher sensitivity in the presence of givinostat (Fig. 1D). These 19 compounds belong to 7 distinct categories: BCL2, FLT3, HSP90, MAPK, PI3K/mTOR, topoisomerase and p53-MDM2 inhibitors. By setting a threshold of DSS = 25 to consider a drug effective, we chose one drug from each category, specifically selecting the compound with the highest DSS score in givinostat condition and the largest difference between givinostat and DMSO conditions for subsequent analyses. The drugs that passed these parameters were the following: venetoclax, luminespib, trametinib, sapanisertib, etoposide, and idasanutlin (Fig. 1E).

### 3.2 Validation of the most promising drug combinations on BCP-ALL and on non-leukemic lymphoblastoid cell lines

**Fig. 2:**
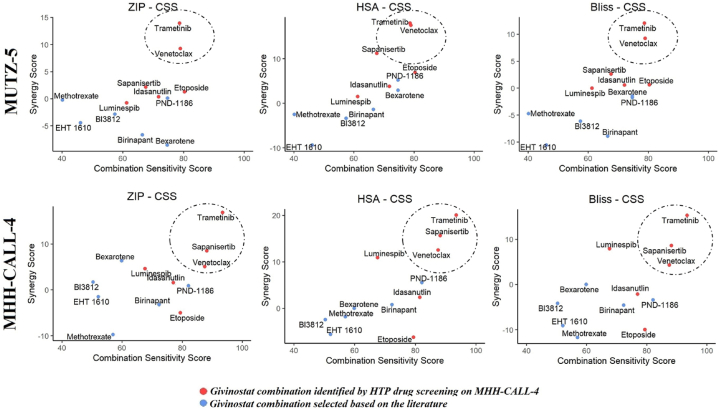
Validation of the identified promising givinostat combinations in CRLF2r BCP-ALL cell lines in a matrix of increasing concentrations Graphs illustrating the degree of interaction (synergy, additivity, antagonism) by 3 popular metrics (y-axis: ZIP, HAS, Bliss) and the combination sensitivity (x-axis: CSS) in the validation setting on MUTZ-5 and MHH-CALL-4. The compounds identified by the HTP screening are highlighted in red, while in blue further compounds included in the validation. The circle encompasses the most promising combination pairs in terms of high synergy and combination sensitivity. The combinations consistently observed in the upper right quadrant of the graph, across all interaction metrics utilized, and in both tested CRLF2r cell lines, were selected for further analysis. (For interpretation of the references to color in this figure legend, the reader is referred to the Web version of this article.)

The 6 most promising partners of givinostat identified by the HTP screening underwent further validation, as well as 6 other compounds (bexarotene, birinapant, methotrexate, and PND-1186, all included in the HTP drug library, as well as BI3812 and EHT 1610) published to be effective as a single treatment against BCP-ALL cases with features frequently co-occurring with CRLF2r (DS-ALL, IKZF1plus or deletion, Ph-like gene expression profile) [[49], [50], [51], [52], [53]]. We validated these compounds *in vitro* by assessing the anti-leukemic activity of the drugs alone or in combination with givinostat against MHH-CALL-4 and MUTZ-5 cell lines, in a matrix-based concentration range of the two drugs. For the drug pairs under investigation, the Cell Titer Glo metabolic assay allows us to estimate both their degree of interaction (synergy, additivity, antagonism) using three common metrics (ZIP, HSA, Bliss), as well as the combination sensitivity (CSS), a parameter that reflects the efficacy achieved under a given synergy (Fig. 2).

As shown in Fig. 2 (dashed circles), the pairs that possessed the highest synergy and combination sensitivity scores in both cell lines, confirmed by all synergy metrics computed, were givinostat-trametinib and givinostat-venetoclax. The combination of givinostat with sapanisertib was shown to be highly synergic and sensitive for the MHH-CALL-4 cell line, but not for the MUTZ-5 cell line, and was therefore excluded from subsequent analyses.

**Fig. 3:**
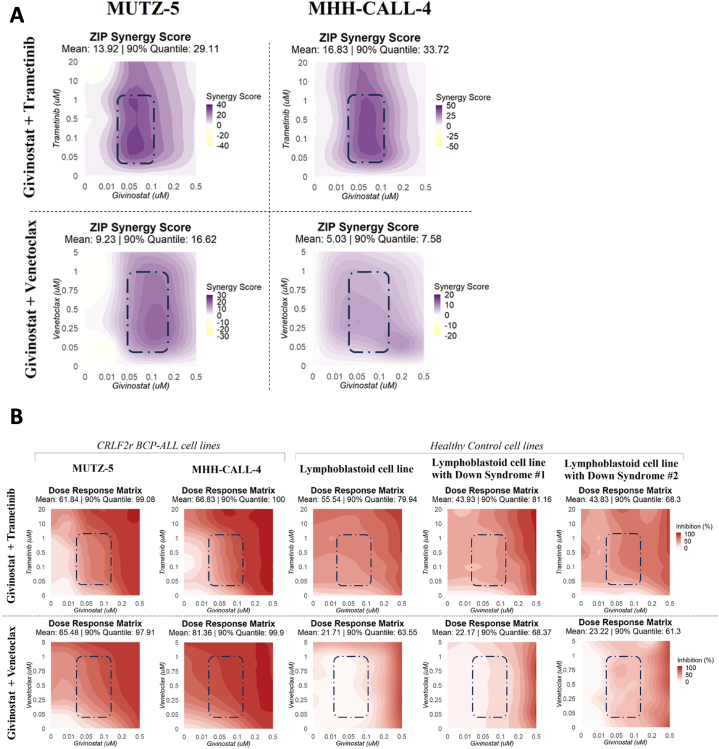
Investigation of the most synergistic and safe concentration range of the promising givinostat combinations tested in leukemic and healthy cell lines A. Heatmaps representing the ZIP synergy score as measured across the tested concentrations of givinostat with trametinib or with venetoclax on MUTZ-5 and MHH-CALL-4. Purple color indicates higher synergy, while white and yellow indicate additivity and antagonism respectively. The most synergistic range of concentrations according to the ZIP metric is highlighted with a dashed rectangle. B. Heatmaps showing the inhibition activity of the identified givinostat combinations for CRLF2r ALL cell lines and non-leukemic controls with or without Down Syndrome. Color intensity reflects the degree of inhibition across the evaluated concentrations, with the most synergistic ranges highlighted with a dashed rectangle. (For interpretation of the references to color in this figure legend, the reader is referred to the Web version of this article.)

In order to identify the range of concentrations of the drugs with the greatest synergistic effect, we analyzed the ZIP synergy score and we observed that the concentrations of givinostat at 0.05–0.1 μM with the second drug at 0.05–1 μM were the ranges with the highest ZIP score for both cell lines (ZIP synergy mean for givinostat plus trametinib: 13.92 and 16.83 for MUTZ-5 and MHH-CALL-4, respectively; and ZIP synergy for givinostat plus venetoclax: 9.23 and 5.03, Fig. 3A). Subsequently, we assessed the inhibitory potency of the two drug combinations in the CRLF2r BCP-ALL cell lines and in a small cohort of non-leukemic lymphoblastoid cell lines derived from healthy children or from children with Down syndrome. As shown in Fig. 3B, within the concentration ranges identified as having the greatest synergy on the leukemic cell lines, we observed high efficacy of these combinations against leukemia, with a lesser effect on healthy cells. In particular, the combination givinostat-venetoclax showed the safest profile.

### 3.3 *Ex vivo* treatment of CRLF2r BCP-ALL patient blasts with the candidate combinations

The selected ranges of drug concentrations showing the greatest synergistic effect in BCP-ALL cell lines were used as a reference for treating *ex vivo* patient blasts with BCP-ALL CRLF2r and positive or not for other characteristics that often co-occur with the rearrangement, such as Down Syndrome, JAK2 mutation and *IKZF1* deletion (see Table 1 for clinical and biological data of the patients).

**Fig. 4:**
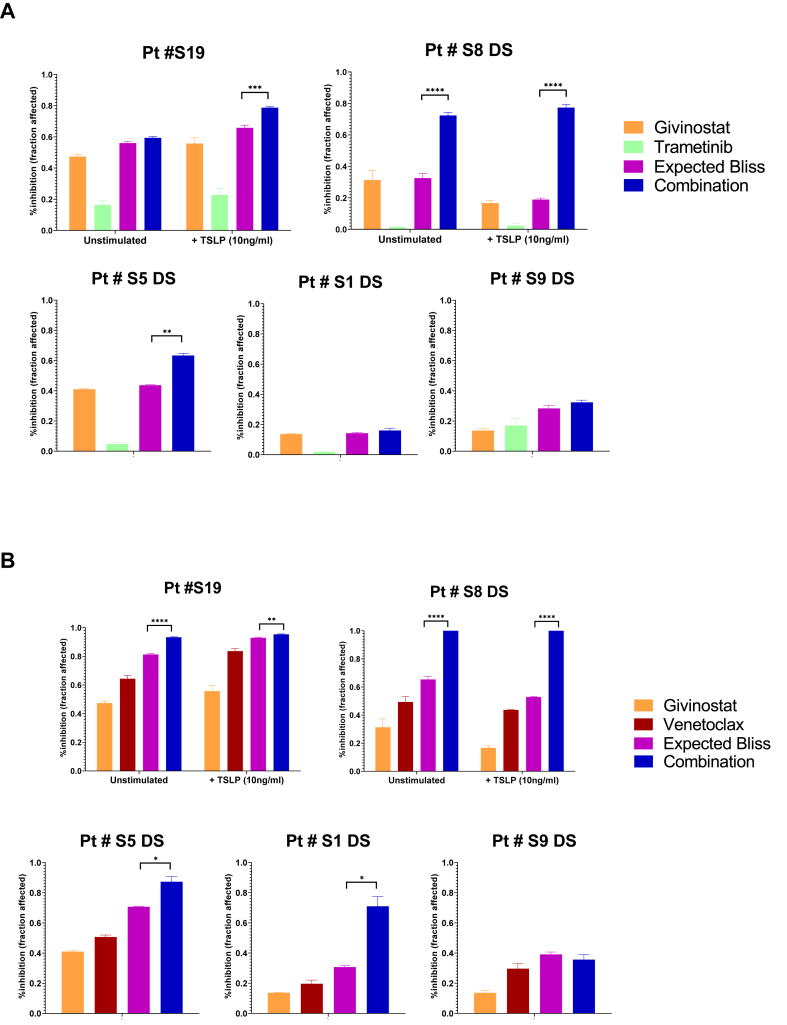
*Ex vivo* validation of candidate givinostat combinations on PDXs A-B. Barplots showing the percent of dead cells of CRLF2r samples upon 72h *ex vivo* culture with givinostat, the partner drug (A: trametinib or B: venetoclax) and their combination, as determined by AnnexinV/7-ADD staining. Data are presented as Mean with Standard Deviation. Welch's *t*-test between the values of the Expected Bliss in case of additivity (purple bars) and the measured observed effect of the combination (blue bars) was applied (*p < 0.05, **p < 0.01, ***p < 0.001; n = 3 replicates per condition). JAK2 wild-type patients (#S19, #S8 DS) are treated in the presence or absence of TSLP 10 ng/ml added concomitantly with the drugs. (For interpretation of the references to color in this figure legend, the reader is referred to the Web version of this article.)

PDX samples of these 5 patients were treated with givinostat, trametinib, and venetoclax as single agents and in combination (givinostat + trametinib and givinostat + venetoclax), using the doses of 0.1 μM for givinostat, 0.5 μM for trametinib and 0.05 μM for venetoclax as a backbone, except Pt #S5 DS for which givinostat was reduced to 0.05 μM and venetoclax to 0.01 μM as the cells were already highly sensitive to these drugs when used alone. After 72h we evaluated the apoptosis by AnnexinV/7-AAD staining using FACS analysis. Interestingly, the observed effect of the combination (expressed in fraction of apoptotic cells) was statistically significant (p-value<0.05) higher than the expected bliss, indicating a synergistic effect in 3/5 patients for givinostat-trametinib and in 4/5 patients for givinostat-venetoclax. In the remaining cases the effect was additive (Fig. 4A and B).

It should be noted that the two patients #S1 DS and #S9 DS in which the drugs showed only an additive effect (2/2 for givinostat + trametinib and 1/2 for givinostat + venetoclax) are both positive for the IKZF1plus characteristic (Fig. 4 and Table 1).

To ensure the evaluation of the effect of the drugs in cells in which the JAK2/STAT5 pathogenic pathway was active, we performed the treatment for the two patients wild-type for JAK2 (Pt #S19 and Pt #S8 DS) also in the presence of the molecule thymic stromal lymphopoietin (TSLP), the ligand of CRLF2 receptor, confirming the efficacy of the combinations even with the pathway activated (Fig. 4 A, B).

### 3.4 Analysis of gene expression perturbations after givinostat treatment supporting the observed drug synergies

We took advantage of gene expression data of 5 CRLF2r BCP-ALL patient-derived xenograft samples (3 out of 5 with DS) treated with vehicle or givinostat *ex vivo* for 6 h that we previously published (study GSE77270) [27] in order to investigate transcriptional modifications caused by givinostat that could support the observed drug synergies.

**Fig. 5:**
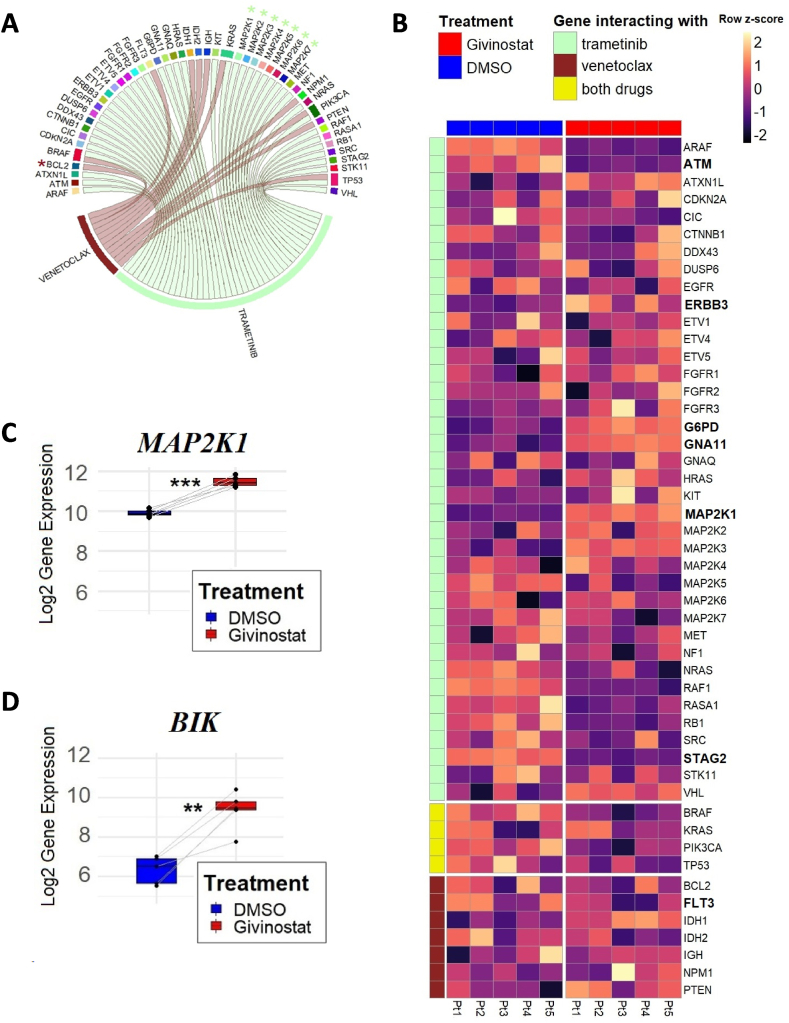
Analysis of the ability of givinostat to perturb the expression of genes known to interact with trametinib or venetoclax based on gene expression microarray data of five givinostat-*ex vivo* treated CRLF2r PDX A. Genes that are known or predicted to interact with trametinib (in green) or with venetoclax (in red) according to the Drug-Gene Interaction database (DGIdb). With * are marked genes with known direct interactions. B. Heatmap of the expression of trametinib or venetoclax interacting genes (rows) between the DMSO (blue) versus givinostat (red) condition in the five CRLF2r PDX (columns). Multiple corresponding Affymetrix probes of the dissected genes were collapsed to single genes by GSEA software. Color intensity represents the row z-score of normalized gene expression and genes among those with a significant differential expression (adjusted p-value <0.05) are marked in bold. C. Boxplot summarizing the expression level of the trametinib direct target *MAP2K1* in the five PDX in DMSO vs givinostat condition. Significance is indicated by the adjusted p-value (***p < 0.001). D. Boxplot of *BIK* gene expression level in DMSO and givinostat condition (adjusted p-value: **p < 0.01). (For interpretation of the references to color in this figure legend, the reader is referred to the Web version of this article.)

Drug-Gene Interaction database (DGIdb) revealed 42 and 11 genes interacting with trametinib and venetoclax respectively, with only 4 of them in common (*BRAF*, *KRAS*, *PIK3CA*, *TP53*) (Fig. 5A). Consequently, we evaluated the expression of those 49 genes across the gene expression profile of the PDX samples treated with DMSO or givinostat (Fig. 5B). Among the 49 genes, 7 were found to be differentially expressed (adjusted p-value<0.05) between the two conditions (Fig. 5B, genes in bold). In particular, 4 genes were upregulated (*ERBB3*, *GNA11*, *G6PD*, *MAP2K1*) and 3 were downregulated (*STAG2*, *ATM,* and *FLT3*) by givinostat. *FLT3* was the only differentially expressed gene associated with venetoclax, whereas all the other genes are known interactors with trametinib. Notably, givinostat was able to upregulate *MAP2K1,* a gene encoding for MEK1 protein kinase which is part of the RAS/MAPK signaling pathway and annotated by DGIdb to be a direct target of trametinib (log2 fold change: 1.45, Fig. 5C). Regarding the direct target of venetoclax, *BCL2*, we did not observe a statistically significant change in expression upon givinostat treatment (Fig. 5B). We then investigated the behavior of genes belonging to the BCL-2 family and observed a marked increase in the expression of the pro-apoptotic gene *BIK* (log2 fold change: 3.03, Fig. 5D).

**Fig. 6:**
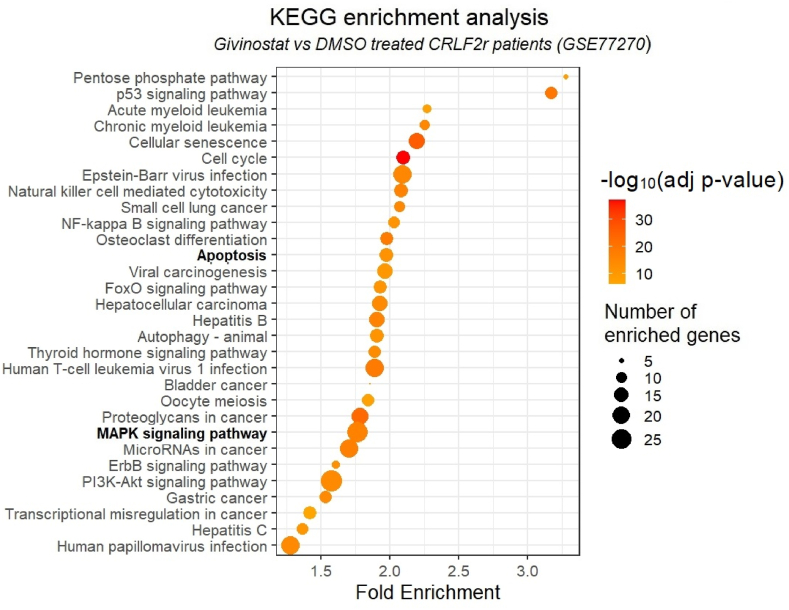
KEGG enrichment analysis for all differentially expressed genes between DMSO and givinostat treatment in the CRLF2r PDX samples Graph summarizing the top 30 pathways based on statistical significance, sorted according to fold enrichment (x-axis) and illustrating their abundance in differentially expressed genes (dot magnitude).

KEGG enrichment analysis of all the differentially expressed genes between PDX CRLF2r BCP-ALL cells treated with DMSO versus givinostat revealed the pathways that were heavily affected by givinostat. Interestingly, both apoptosis and MAPK signaling pathways were significantly enriched (Fig. 6).

## 4 Discussion

This study has provided a model for identifying effective anti-leukemic drug combinations for CRLF2r BCP-ALL, a subgroup characterized by poor outcome [24,54] and frequently represented in pediatric ALL patients with Down Syndrome [17,18,26]. Starting from our previous observations demonstrating the high efficacy of the HDAC inhibitor givinostat against this subtype of leukemia [27], we investigated possible synergism with other drugs. To achieve this purpose, we applied a drug repurposing strategy, performing a high-throughput screening with a library comprising 174 compounds, FDA/EMA-approved or in preclinical studies, already successfully used in other studies [26,38]. This approach revealed that the combinations of givinostat with trametinib and with venetoclax were highly synergistic and effective on CRLF2r ALL cell lines and primary blasts. We identified the concentration ranges possessing the highest synergic score and the safest profile in *in vitro* experiments testing lymphoblastoid cells derived from non-leukemic children with or without Down Syndrome as a control.

Since *CRLF2* rearrangement alone causes overexpression of the receptor, but in the absence of additional JAK2 or CRLF2 activating mutations the signaling is described as still ligand-dependent [55], for patients wild-type for the mutations we tested the efficacy of the selected drug combinations even in the presence of TSLP. Our results indicate that the proposed targeting strategy has the potential to target CRLF2r leukemic cells regardless of their dependency on TSLP, therefore in the presence or absence of additional mutations causing the constitutive activation of the pathway.

Importantly, 4 out of the 5 tested primary blasts were derived from patients affected by Down Syndrome, a very fragile subgroup that suffers from frequent relapses and high therapy-related toxicity. The drug combinations identified in this study may represent an important weapon for this cohort since *CRLF2* is altered in about 60 % of DS-ALL [26] and we showed that, at nanomolar concentrations, givinostat plus trametinib and givinostat plus venetoclax were able to kill DS-ALL CRLF2r cells, while exhibiting reduced efficacy on DS non-leukemic cells. Particularly, the givinostat-venetoclax combination showed the most favorable safety profile. Here we tested the non-toxicity of the drugs on lymphoblastoid lines *in vitro*, but further studies will be needed to confirm our observations in more comprehensive models.

Finally, our representative PDX cohort also included two CRLF2r ALL patients belonging to the IKZF1plus subgroup, which represents a category of patients with a very poor outcome due to defined drug resistance [56,57]. These patients were found to be highly resistant to treatment also in our study, showing the least responsive profile to the identified givinostat combinations, with the exception of one of the two cases very sensitive to givinostat plus venetoclax. Further studies are needed to understand these differences and to identify targeted drug combinations for these difficult-to-treat patients.

Efforts on the therapeutic management of pediatric ALL through epigenetic drugs are already well documented [58], and HDAC inhibitors have been incorporated in combination strategies with various drug categories for the treatment of cancer at preclinical or clinical stages [59,60]. Among these approaches, drug combinations with givinostat were evaluated for several non-leukemic tumors [[61], [62], [63], [64]].

Here we reported for the first time a synergistic interaction of givinostat with trametinib, an approved MAPK inhibitor [65], and with venetoclax, an approved BCL-2 inhibitor [66], in CRLF2r BCP-ALL, a subgroup for which givinostat had previously proven to be an interesting candidate [27]. However, it's important to underline that we cannot exclude the possibility that these combinations might also be effective in other subtypes of BCP-ALL and tumors.

Our findings are further supported by the observed interference of givinostat with MAPK signaling in melanoma cells [67], in hepatic stellate cells [68], and in Hodgkin lymphoma cells [64]. Similarly, givinostat has been reported to affect the apoptosis pathway in various diseases [69,70]. Finally, the synergistic effect of combining HDAC inhibition with MAPK or BCL-2 inhibition has already been successfully described in multiple other settings [[71], [72], [73], [74], [75]].

In this study, we investigated the gene expression changes induced by givinostat [27] in order to dissect the mechanisms underlying the observed drug synergies. We extracted 49 genes known to interact with trametinib or venetoclax and investigated them in the gene expression profile dataset of CRLF2r BCP-ALL blast cells in the presence or absence of givinostat. Among these genes, *FLT3* was the only one associated with venetoclax, though indirectly, proven to be differentially expressed after givinostat treatment. Interestingly, FLT3 inhibition has been reported to increase venetoclax response in AML settings [76,77]. The direct target of venetoclax, *BCL2*, was not present in the list of genes significantly modified by givinostat. Given that the sensitivity to venetoclax is not only determined by its target molecule BCL-2 alone but rather is affected by different family members [78], we investigated the ability of givinostat to modulate other members of the BCL-2 family and we found a strong upregulation of the pro-apoptotic gene *BIK*. Interestingly, it is described that drugs that upregulate BH3-only proteins such as BIM and BIK could potentiate the effects of venetoclax in hematopoietic malignancies [79]. With regards to genes associated with trametinib, we observed the upregulation of *MAP2K1*, the gene encoding its direct target MEK1 [80]. We can therefore speculate that this modulation can make cells more sensitive to the action of this specific inhibitor. Further studies are needed to confirm the role of these gene expression modifications in the drug synergies and, more broadly, to elucidate the underlying mechanism of action of these combinations.

In conclusion, in the present study we applied a drug repurposing strategy by performing HTP screening with a library of drugs already approved or in preclinical studies to investigate possible synergies with the HDAC inhibitor givinostat, a drug already proven to cause the inactivation of JAK/STAT signaling network and to induce CRLF2 positive leukemic cell death. With this approach, we showed the strong anti-leukemic potential of co-targeting the JAK2/STAT5 pathway via givinostat together with the MAPK signaling via trametinib or the apoptotic signaling via venetoclax. Thus, these two combination treatment options are worthy of further investigation in the treatment of CRLF2r ALL pediatric patients, a subgroup that urgently requires new therapeutic strategies.

## Ethics approval and consent to participate

The investigation was conducted in accordance with the ethical standards of the Declaration of Helsinki, and with national and international guidelines. We obtained written informed consent from patients aged 12 and older and from their parents or legal representatives. Animal testing has been conducted in accordance both with the current European and National Legislation (authorization n◦ 09/2018, protocol FB7CC.38 released by the Italian Ministry of Health) in compliance with the Animal Welfare Organisation of the University of Milano-Bicocca.

## Funding

This work was supported by the 10.13039/501100003196Italian Ministry of Health, grant Ricerca Finalizzata-Giovani Ricercatori (GR-2016-02364753 to 10.13039/100023333CP, GF and MB) and by “Comitato Maria Letizia Verga”. AO acknowledges the funding from the European Union's 10.13039/501100007601Horizon 2020 research and innovation programme under the Marie Skłodowska-Curie grant agreement No 813091 (ARCH, Age-Related Changes in Hematopoiesis). SB acknowledges the financial support from the 10.13039/501100001659Deutsche Forschungsgemeinschaft (10.13039/501100001659DFG, 10.13039/501100001659German Research Foundation) −270650915 (Research Training Group GRK 2158, 10.13039/501100017484TP 2d to S.B.) and Elterninitiative Kinderkrebsklinik e.V. LV was supported by the Doctoral Program in Molecular and Translational Medicine (DIMET, 10.13039/501100002954University of Milano-Bicocca).

## Data availability statement

All microarray raw data (CEL files) and probe set signals are available at the National Center for Biotechnology Information Gene Expression Omnibus database (GEO, http://www.ncbi.nlm.nih.gov/geo/), series accession number GSE77270 (http://www.ncbi.nlm.nih.gov/geo/query/acc.cgi?token=mdwpqoeqhjuhzef&acc=GSE77270) [27].

## CRediT authorship contribution statement

**Athanasios Oikonomou:** Writing – original draft, Visualization, Validation, Software, Methodology, Investigation, Formal analysis. **Titus Watrin:** Visualization, Methodology, Investigation, Formal analysis. **Luigia Valsecchi:** Methodology, Formal analysis. **Katerina Scharov:** Methodology, Formal analysis. **Angela Maria Savino:** Methodology, Formal analysis. **Julian Schliehe-Diecks:** Methodology, Formal analysis. **Michela Bardini:** Writing – review & editing, Funding acquisition. **Grazia Fazio:** Writing – review & editing, Funding acquisition. **Silvia Bresolin:** Writing – review & editing, Methodology, Formal analysis. **Andrea Biondi:** Supervision, Resources. **Arndt Borkhardt:** Supervision, Resources. **Sanil Bhatia:** Supervision, Resources. **Giovanni Cazzaniga:** Supervision, Resources. **Chiara Palmi:** Writing – review & editing, Supervision, Funding acquisition, Conceptualization.

## Declaration of competing interest

The authors declare that they have no known competing financial interests or personal relationships that could have appeared to influence the work reported in this paper.
